# Bleeding pattern after medical management of early pregnancy loss with mifepristone–misoprostol and its prognostic value: a prospective observational cohort study

**DOI:** 10.1007/s00404-021-06291-5

**Published:** 2021-10-25

**Authors:** Simon-Hermann Enzelsberger, Daniela Wetzlmair, Philipp Hermann, Helga Wagner, Omar Shebl, Peter Oppelt, Philip Sebastian Trautner

**Affiliations:** 1 Department of Gynecology, Obstetrics and Gynecological Endocrinology, Kepler University Hospital, Johannes Kepler University Linz, Altenberger Strasse 69, 4040 Linz, Austria; 2 Department of Gynecology and Obstetrics, Pyhrn-Eisenwurzen Klinikum, Sierninger Straße 170, 4400 Steyr, Austria; 3 Center for Clinical Studies, Johannes Kepler University Linz, Altenberger Strasse 69, 4040 Linz, Austria; 4 Institute of Applied Statistics, Johannes Kepler University Linz, Altenberger Strasse 69, 4040 Linz, Austria

**Keywords:** Early pregnancy loss, Miscarriage, Mifepristone, Misoprostol, Bleeding pattern

## Abstract

**Purpose:**

To improve counseling of women by reporting bleeding characteristics at home after medical management of an early pregnancy loss (EPL) with mifepristone and misoprostol, and to evaluate occurring bleeding patterns as a prognostic tool.

**Methods:**

This prospective two-center observational cohort study enrolled 197 women who presented with an EPL (embryonic or anembryonic miscarriage) from December 2017 to April 2019 and chose a home-based medical management with 200 mg mifepristone and 800 mcg misoprostol. From the day of mifepristone intake, the strength of vaginal bleeding was recorded daily for 2 weeks by the patient herself using a diary sheet. Treatment success was defined as no histologically confirmed retained products of conception (RPOC) within 3 months. After considering all drop-out criteria, 154 women were included in the analysis.

**Results:**

40.0% of patients (95% CI 30.4–49.6) already reported bleeding onset in the time period between the intake of mifepristone and misoprostol. The median duration of vaginal bleeding including spotting was 13 days. The chance of RPOC was about sixfold (OR 6.06, 95% CI 2.15–17.10) in the group of persistent bleeding after 2 weeks compared to the group with a terminated bleeding at that time. Exploratory regression analysis indicated association of higher serum levels of leukocytes at treatment start with RPOC (*p* = 0.013).

**Conclusions:**

Terminated bleeding after 2 weeks is a useful indicator for successful medical induction of EPL. Women undergoing medical treatment with mifepristone must be informed about the high frequency of bleeding onset before misoprostol intake.

**Clinical trial registration:**

DRKS—German Clinical Trials Register, ID: DRKS00013515, registration date 05.12.2017. http://www.drks.de/DRKS00013515.

**Supplementary Information:**

The online version contains supplementary material available at 10.1007/s00404-021-06291-5.

## Introduction

Early pregnancy loss (EPL) occurs in up to 30% of pregnancies after successful implantation [1]. Due to technical advances in transvaginal sonography, EPL is diagnosed more and more often before clinical symptoms or spontaneous expulsion occur (so-called missed miscarriage). In this situation there are three accepted treatment options: the expectant management (waiting), the surgical procedure (dilatation and curettage–D&C) or the medical induction [2, 3]. The medical management offers a possibility for women who on the one hand want an active management and on the other hand want to avoid a surgical intervention. Numerous trials have shown that initiating with the prostaglandin E1 analog misoprostol is a safe and effective method [4–7]. Recent data recommend the additional use of mifepristone to further improve success [8, 9]. Nevertheless, existing information about the combination of mifepristone and misoprostol in the situation of an EPL is still rare. Especially facts about occurring bleeding patterns and their interpretation are missing.

The purpose of this observational study is to improve counseling of patients by reporting bleeding characteristics at home after a combination of mifepristone and misoprostol, and to evaluate occurring bleeding patterns as a prognostic tool for successful medical management.

## Methods

This prospective observational study includes women who presented with an EPL (embryonic or anembryonic miscarriage) from December 2017 to April 2019 at the two participating centers (Kepler University Hospital, Pyhrn-Eisenwurzen Klinikum) and chose a medical management after being consulted about all possibilities. The participation was limited to a crown-rump-length of 60 mm in the transvaginal sonography. Incomplete miscarriage (gestational sac already expulsed) and inevitable miscarriage (low-lying gestational sac and vaginal bleeding) as well as multiple pregnancies were excluded.

After informed consent and study inclusion, the patients took 200 mg mifepristone (Mifegyne®) orally under medical supervision. If the mother was rhesus negative, 1500 I.U. (300 mcg) anti-D-immunoglobulin (Rhophylac®) was additionally administered. Misoprostol medication (Cyprostol®) was then handed over to the women and an appointment was made after 14 days (± 2 days). At home, after 36–48 h (individual time period tailored to the time of day of mifepristone intake), the patients took 400 mcg misoprostol and repeated the intake three hours later (all together 800 mcg misoprostol, buccal or sublingual administration).

At baseline, investigators conducted a physical examination, a transvaginal ultrasound, and obtained a blood sample (human chorionic gonadotropin—hCG, progesterone, blood count, and blood group). From the day of mifepristone intake (day 1), strength of vaginal bleeding was recorded daily by the patient herself using a diary sheet, which was given to her in paper at the time of study enrollment. For each day, participants indicated in three categories whether bleeding was ‘heavy or moderate’, ‘light or spotting’ or ‘none’. Participants selected the category of bleeding without guidance or definition by study staff. At the follow-up appointment after 14 days (± 2 days), the completed bleeding diary was collected. Based on symptoms and an ultrasound scan, further measures were counseled (eg more misoprostol tablets or D&C) or a check-up after the next menstrual bleeding was advised. Each patient with sonographic signs of retained products of conception (RPOC) and/or bleeding problems could decide for herself whether she would like to continue with a wait-and-watch management, further misoprostol tablets or surgery; decision was followed by individual follow-up appointments. If no operative intervention was performed up to three months after mifepristone intake and there were no persistent clinical or sonographic signs of RPOC, follow-up for patient was closed.

Treatment success was defined as no histologically confirmed RPOC in a D&C within three months after mifepristone intake. In addition, sonographic evidence of RPOC at the control appointment was evaluated and classified into three categories (‘no signs of RPOC’, ‘questionable signs of RPOC’ and ‘clear signs of RPOC’). STROBE checklist was used for reporting [10].

### Statistical analyses

The sample size was convenience-based: We used previous data from one study center to predict the number of EPL-patients with medical treatment during 1 year. Bleeding duration was calculated as difference of days between the first bleeding-free day and the day of bleeding onset (both data are provided by the bleeding diary). To take into account right-censoring of bleeding duration due to persistent bleeding at control appointment, the duration of bleeding was analyzed using a Kaplan–Meier curve. To investigate effects of baseline variables on the duration of bleeding, Cox proportional hazards models were fitted. Variable selection was performed backward retaining variables with *p*-value < 0.1 in the final model. Additionally, logistic and ordered logistic regression analyses were conducted to scan for predictive factors of treatment success (modeling effects of bleeding parameters as well as selected biological and demographic variables on the probability of overall and sonographic success, i.e. no signs of RPOC, using a stepwise selection procedure based on the Akaike Information criterium). Missing values were not replaced. Data analysis was performed using the statistics software R (2020) [11] {Team, 2020 #135}.

### Ethical approval

The study was approved by the institutional ethics committee of the Johannes Kepler University (submission number C-142-17, date of approval 21.11.2017) and registered in the German Clinical Trials Register (ID: DRKS00013515, http://www.drks.de/DRKS00013515, registration date 05.12.2017, date of initial participant enrollment 20.12.2017).

## Results

**Fig. 1:**
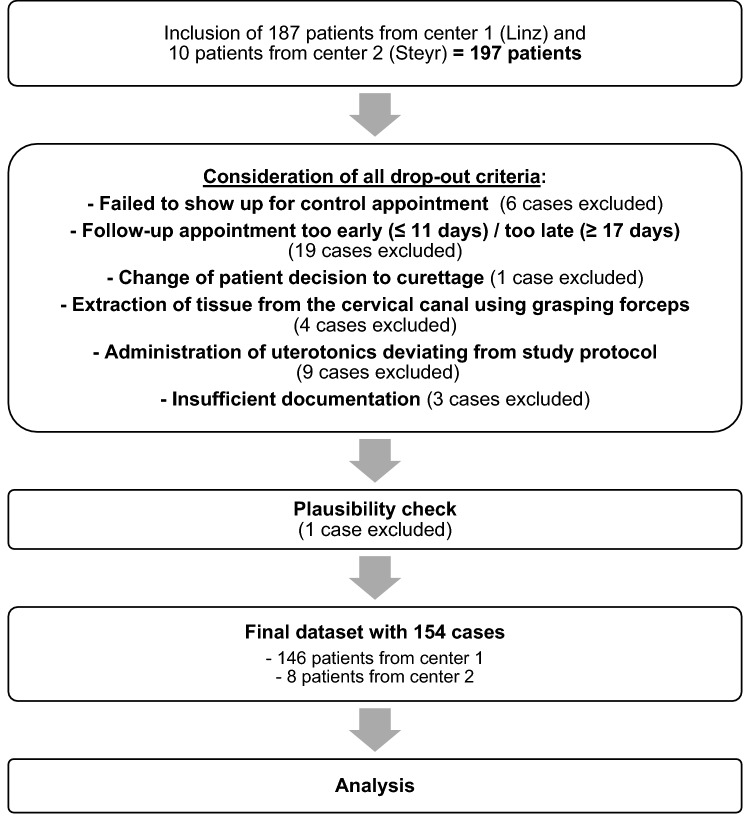
Origin of data set

197 women were enrolled in the trial. After taking into account all drop-out criteria, the number of cases was reduced to 155. The reasons for the drop-outs are listed in Fig. 1. To eliminate incomplete information and inconsistencies, the information in the reports and in the stored ultrasound images were compared and subjected to a plausibility check. This led to removal of one patient with divergent measurement values in the source data. Altogether, the final data set contains 154 cases, including 144 women with embryonic miscarriage (93.5%) and 10 women with anembryonic miscarriage (6.5%).

Characteristics of participants are provided in Table 1. The median calculated gestational age based on the last menstruation is the 10th pregnancy week; the median sonographically-determined pregnancy week is the 7th week according to a median crown-rump-length (CRL) of 6 mm. At enrollment, 17.8% of the women reported lower abdominal pain and 27.6% reported light bleeding or spotting during the 24 h prior to treatment. Most participants (89.6%) completed their bleeding diaries. The 16 women not returning their diary were excluded from the analysis of data provided only by the diary.

**Table 1:** Characteristics of participants

Variable	*n*	Min	Max	Median (Q1–Q3)
Maternal age [years]	154	18	43	31 (27–36)
Body weight [kg]	137 (17 missing)	43	127	63 (57–73)
BMI [kg/m^2^]	137 (17 missing)	17,22	45,70	22.91 (20.90–26.47)
Gravidity [*n*]	151 (3 missing)	1	10	2 (1–3)
Parity [*n*]	151 (3 missing)	0	6	0 (0–1)
Previous miscarriages [*n*]	149 (5 missing)	0	3	0 (0–0)
Previous induced abortions [*n*]	149 (5 missing)	0	5	0 (0–0)
Pregnancy age based on the last menstruation [days]	148 (6 missing)	45	93	66 (62–72)
Crown-rump-length (CRL) [mm]	133 (21 missing)	0	26	6 (4–11)
Difference between the ultrasound-determined and the calculated pregnancy week based on the last menstruation [weeks]	119 (35 missing)	0	6	3 (2–4)
Human chorionic gonadotropin (hCG) [IU/L]	144 (10 missing)	613	165,114	23,970 (8838.50–42,876.25)
Leukocytes [G/L]	143 (11 missing)	4.60	16.50	8.20 (7.10–9.30)
Time interval between mifepristone intake and control appointment [days]	154	12	16	14 (14–15)

*Min* Minimum, *Max* Maximum, *Median (Q1–Q3)* Median (first quartile to third quartile)

In 116 of the 154 included study participants (75.3%) a medical induction with the described mifepristone-misoprostol regimen was carried out without performing a D&C within three months. When patients with a D&C but without histological proof of RPOC (*n* = 12) were additionally counted to the successful cases (as defined in this study), an overall success rate of 83.1% (*n* = 128) was achieved. In the 38 cases with D&C, only two women (1.3% of total cases) had the indication for surgery because of a not expelled gestational sac. In the remaining 36 women, who were also indicated for D&C, sonographically-detected RPOC and/or bleeding disorders were documented. At the scheduled follow-up visit after 2 weeks, 48 patients decided to take additional misoprostol doses due to sonographic signs of incomplete expulsion. Half of these women (*n* = 24) did not receive the goal of a complete expulsion and finally needed a surgical intervention. There were no signs of gestational trophoblastic disease in the found RPOC.

When looking at the sonography results at the control appointment, 45.5% (*n* = 70) of the examinations were classified as ‘no signs of RPOC’ and 31.8% (*n* = 49) as ‘clear signs of RPOC’. The remaining 22.7% (*n* = 35) of the scans showed questionable signs of incomplete expulsion (not clearly classifiable). Only three women showed a persistent gestational sac at the control appointment (1.9% of all 154 included women). The median measured distance between the two myometrium layers in a uterine sagittal plane–often referred to as endometrial thickness–was 9 mm (Q1–Q3: 6–12.5). When only looking at the subgroup of women with histological proof of RPOC at end of study, the measured endometrial thickness increases to a median of 15 mm (min 9 mm, max 31 mm, Q1–Q3: 12.5–17.5).

The observed bleeding characteristics in the first 2 weeks from the day of mifepristone intake are summarized in Table 2. The highest proportion of participants (45.3%) had the onset of vaginal bleeding at day 3, which coincided with misoprostol application. The remaining participants reported their bleeding onset in the time period between mifepristone and misoprostol intake (28.8% on day 1 and 25.9% on day 2; together 54.7%). When discounting patients with a light bleeding or spotting in the 24 h prior the mifepristone intake, still 40.0% (95% CI 30.4–49.6) of remaining women reported a vaginal bleeding before the third day with misoprostol application. There were no participants reporting a bleeding onset later than day 3.

**Table 2:** Bleeding characteristics

Variable (categorical)		Absolute frequency	Percentage of valid
Onset of vaginal bleeding (*n* = 139, 15 missing)	Day 1	40	28.8%
	Day 2	36	25.9%
	Day 3	63	45.3%
Onset of vaginal bleeding before misoprostol (except women with a reported bleeding before mifepristone) (*n* = 100)	No	60	60.0%
	Yes	40	40.0%
Bleeding status at control appointment (*n* = 153, 1 missing)	Persistent bleeding	73	47.7%
	Terminated bleeding	80	52.3%
Stronger bleeding after already spotting or no bleeding (*n* = 137, 17 missing)	No	105	76.6%
	Yes	32	23.4%

Variable (metric)	Min	Max	Median (Q1–Q3)
Number of days with heavy/moderate bleeding [days] (*n* = 136, 18 missing)	1	13	5 (4–7)
Number of days with spotting [days] (*n* = 136, 18 missing)	0	13	5 (4–7)

Predictive value of bleeding persistence				
		Histologically proven RPOC	No histological proof of RPOC	Odds ratio (95% CI)
Bleeding status at control appointment (*n* = 153, 1 missing)	Persistent bleeding	21 (28.8%)^a^	52 (71.2%)^a^	6.06 (2.15—17.10)
	Terminated bleeding	5 (6.25%)^a^	75 (93.75%)^a^	

^a^Conditional relative frequencies in parenthesis

**Fig. 2:**
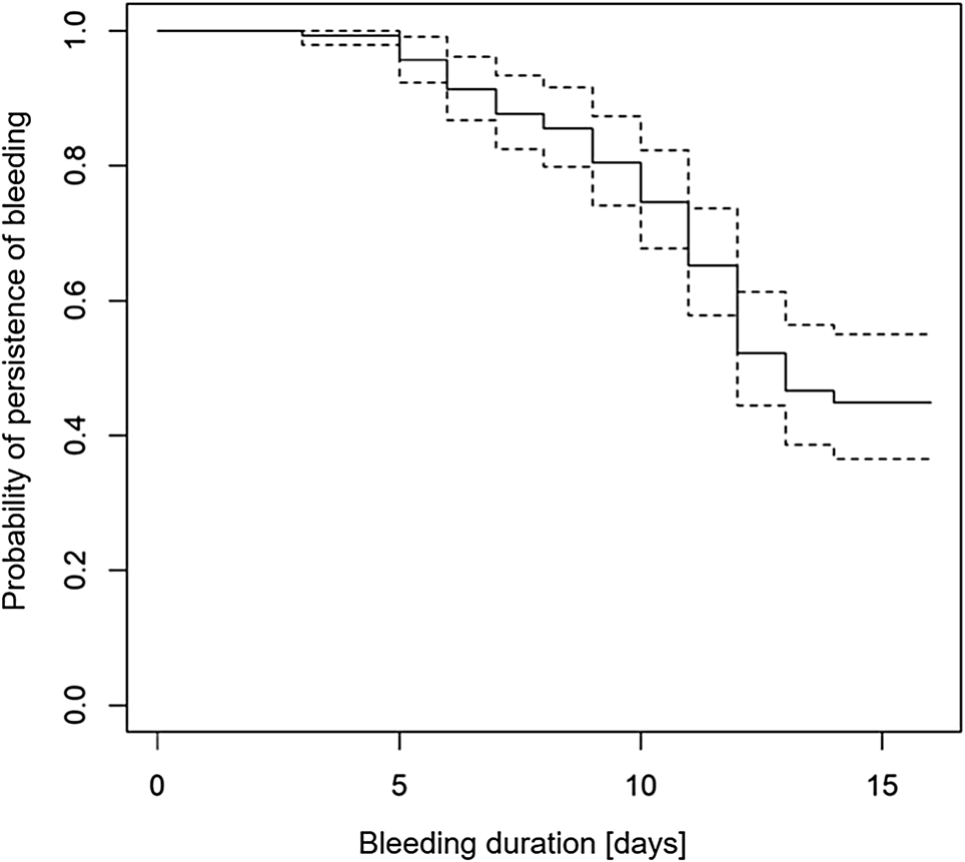
Bleeding duration (Kaplan–Meier curve)

To analyze the bleeding duration, we computed a Kaplan–Meier curve (Fig. 2). The median survival time (= duration) of vaginal bleeding including spotting was 13 days, which means that after 13 days the bleeding in half of our patient cohort had stopped. In the Cox proportional hazard model several potential influencing factors on the hazard of terminated bleeding were considered. After backward variable selection, two variables remained in the model with significant effects: Higher values of HCG (hazard ratio (HR): 0.862; *p* = 0.025) and higher values of leukocytes (HR: 0.845; *p* = 0.027) decrease the hazard of bleeding termination, which corresponds to an increased bleeding duration.

The median duration of reported ‘heavy or moderate bleeding’ was 5 days (Q1–Q3: 4–7); the same time span was shown for the number of spotting days (median 5 days, Q1–Q3: 4–7). 23.4% of the participants noted a stronger bleeding after already reducing to spotting or even a bleeding-free day.

If vaginal bleeding persisted until the control appointment (usually after 14 days), 28.8% of these women finally had a D&C with histologically confirmed RPOC. On the other hand, only 5 of 80 women with a terminated bleeding (6.25%) ended up with histological proven RPOC. Therefore, the odds ratio of histological proven RPOC of women presenting with a persistent bleeding at the control appointment is 6.06 (95% CI 2.15–17.10) compared to the terminated bleeding group. Moreover, 84.9% of the women with a persistent bleeding at the control appointment showed abnormalities in the ultrasound examination (53.4% with clear signs of RPOC and 31.5% with at least questionable signs of RPOC). No patient with a terminated bleeding and missing signs for RPOC on ultrasound after 14 days needed a D&C during the follow-up period of three months.

Additionally, binary and ordinal logistic regression analyses were performed to scan for predictive factors of overall and sonographic success. The results for each regression model can be found in detail as supporting information (Table S1 and S2). In both models, effects of ‘persistent bleeding at control appointment’ (*p* = 0.007 and *p* = 0.048) as well as leukocytes (*p* = 0.013 and *p* < 0.001) were negative and significant–indicating a lower chance of success.

## Discussion

Women routinely have some uterine bleeding after the expulsion of an EPL, so it is always a challenge to distinguish normal from abnormal bleeding. To date, no trial with the primary endpoint of describing bleeding patterns after medical management of EPL with the recommended drug combination of mifepristone and misoprostol is known to the authors. The now available results of this study should provide the necessary facts to better counsel affected women.

The majority of included patients (54.7%) experienced a vaginal bleeding before the contractive effect of misoprostol. Even when discounting women with light bleeding or spotting before mifepristone intake, still 40.0% of patients in the remaining subgroup reported a bleeding before the third day with the misoprostol application. The current literature lacks facts about proportions of bleeding onset in the time interval between mifepristone and misoprostol. Especially in the field of missed miscarriage, we were not able to find any studies on occurring bleeding rates before misoprostol use. When extending the search to the field of induced abortion, the existing evidence is rising. De Nonno et al. reported onset of bleeding in 21% of patients before misoprostol use (using 200 mg mifepristone) [12]. To complete the picture, we were also searching for spontaneous expulsion rates of missed miscarriages (without medical treatment), but we could not find any reported rates within 48 h after the diagnosis.

Another point of interest of this study was the meaningfulness of persistence of vaginal bleeding for more than 2 weeks. The chance of histological proven RPOC was about sixfold in the group of women with persistent bleeding after 2 weeks compared to those with terminated bleeding at that time. This is an important information for both the patient and the treating physician. Previous studies of women with medical induction of an EPL reported a mean of 9 to 16 bleeding days [9, 13, 14] without forming groups according to success. The estimated median bleeding duration in our study was 13 days.

Many (but rather small) studies have already tried to identify predictive factors of success for the medical management of an EPL. So far, no clear predictive factors were found. As an example, a recent study by Sonalkar et al. was not able to detect any clinical predictors of treatment success [15]. In our study, we performed exploratory regression analyses and found significant effects of serum level of leukocytes (higher levels increasing the risk of RPOC). The above shown association of leukocyte levels and bleeding duration could therefore just be a surrogate parameter for overall success. We want to emphasize that none of the participants showed clinical signs of infection at study enrollment. The authors did not find any publication on this possible influencing factor in the medical management of an EPL, so this would be an interesting topic to study.

The strength of this study lies in the prospectively collected data regarding bleeding characteristics in the first 2 weeks after medical management, including also the time span between mifepristone and misoprostol administration. In addition, choosing histological confirmed RPOC as definition of treatment failure represents a clearer endpoint than the often highly subjective indication for D&C.

When interpreting the results, some limitations of the study should be considered:

- After the two-week study period each patient with sonographic signs of RPOC and/or bleeding disorders could decide for herself whether she would like to have a wait-and-watch management, further misoprostol tablets or surgery. Therefore, it is possible that some cases with documented histologically proven RPOC and early D&C might have resolved spontaneously if they had chosen a more expectative approach.
- The overall success rate in this study (83.1%—defined as no histologically confirmed RPOC in a D&C within three months) was comparable to success rates in other recent prospective studies (83–85%—defined as no surgical intervention to complete the miscarriage up to discharge) [9, 16]. However, it is mentionable that the overall D&C-rate was 24.7%, which shows on the one hand the known difficulties in interpreting sonographic results after incomplete expulsion [17], but on the other hand maybe as well that persistent decidual tissue can cause bleeding disorders and pain [18].
- It is important to keep in mind that the day of bleeding onset is not necessarily the same as the day of the expulsion of the pregnancy. In this study, the day of expulsion was not recorded by the patient at home. Therefore, the duration of bleeding might be biased and longer if bleeding start is already before the actual day of expulsion.
- Quite deliberately, to record real-life-effects, it was decided not to train the participants on specific definitions of the different bleeding categories (‘strong/moderate’, ‘light/spotting’, ‘none’). This may have led to different category interpretations by the women themselves. We have chosen a small number of categories (only three) to reduce this possible bias.
- The misoprostol tablets were taken at home and not under medical supervision, so the correct intake by each patient cannot be proven.

## Conclusion

Terminated bleeding after 2 weeks is a useful indicator for successful home-based medical management of an EPL. The chance of RPOC was about sixfold in the group of persistent bleeding after 2 weeks compared to the group with a terminated bleeding at that time. Furthermore, the authors find it important that women should be informed about the high frequency of bleeding onset in the time period between mifepristone and misoprostol intake (about 40%). The influence of serum levels of leukocytes on the success rate needs to be further analyzed.

## Supplementary Information

Below is the link to the electronic supplementary material.

Supplementary file 1: Table S1. Logistic regression model for overall success (selected model after stepwise variable selection based on AIC). Table S2. Ordered logistic regression model for sonographic success (selected model after stepwise variable selection based on AIC).

## Data Availability

The data that support the findings of this study are available from the corresponding author upon reasonable request. The data are not publicly available due to privacy or ethical restrictions.
