# Supplementary material for: Bleeding pattern after medical management of early pregnancy loss with mifepristone–misoprostol and its prognostic value: a prospective observational cohort study
Source: Arch Gynecol Obstet. 2021 Oct 25;306(2):349–55. doi: 10.1007/s00404-021-06291-5 (PMC9349076; doi:10.1007/s00404-021-06291-5)
Supplement: Supplementary file 1 — Table S1. Logistic regression model for overall success (selected model after stepwise variable selection based on AIC). Table S2. Ordered logistic regression model for sonographic success (selected model after stepwise variable selection based on AIC). [file 404_2021_6291_MOESM1_ESM.docx]

**Supporting Information:**

**Table S1. Logistic regression for overall success (selected model after stepwise variable selection based on AIC)**

| **Variable** | **Estimated Coefficient** | **Exp(Estimated Coefficient)** | **Standard Error** | **p-value** |
| --- | --- | --- | --- | --- |
| Persistent Bleeding | -zei1.928 | 0.146 | 0.715 | 0.007 |
| Serum Progesterone | -0.068 | 0.934 | 0.045 | 0.132 |
| Progesterone Intake (y/n) | 4.616 | 101.100 | 3.923 | 0.239 |
| Crown-Rump-Length | 0.160 | 1.173 | 0.069 | 0.021 |
| Leukocytes | -0.385 | 0.680 | 0.155 | 0.013 |

**Table S2. Ordered logistic regression model for sonographic success (selected model after stepwise variable selection based on AIC).**

| **Variable** | **Estimated Coefficient** | **Exp(Estimated Coefficient)** | **Standard Error** | **p-value** |
| --- | --- | --- | --- | --- |
| Persistent Bleeding | -1.140 | 0.320 | 0.576 | 0.048 |
| Days with strong bleeding | -0.267 | 0.766 | 0.133 | 0.044 |
| Days with weak bleeding | -0.241 | 0.786 | 0.127 | 0.059 |
| Stronger bleeding after already spotting or no bleeding | 0.964 | 2.623 | 0.586 | 0.100 |
| Serum Progesterone | -0.072 | 0.931 | 0.059 | 0.223 |
| Progesterone intake (y/n) | 1.532 | 4.626 | 0.794 | 0.054 |
| Leukocytes | -0.468 | 0.626 | 0.129 | < 0.001 |
